# Isolated compression of the ulnar motor branch due to carpal joint ganglia: clinical series, surgical technique and postoperative outcomes

**DOI:** 10.1007/s00590-020-02807-y

**Published:** 2020-10-17

**Authors:** Michela Saracco, Rocco Maria Panzera, Barbara Merico, Francesca Madia, Antonio Pagliei, Lorenzo Rocchi

**Affiliations:** 1 Department of Orthopaedics - Hand Surgery Unit, Fondazione Policlinico Universitario A. Gemelli IRCCS, Roma - Università Cattolica del Sacro Cuore, Largo F. Vito 1, 00168 Rome, Italy; 2 Department of Neurophysiology, Fondazione Policlinico Universitario A. Gemelli IRCCS, Roma - Università Cattolica del Sacro Cuore, Largo F. Vito 1, 00168 Rome, Italy

**Keywords:** Ulnar nerve, Entrapment, Motor branch, Motor dysfunction, Joint ganglia

## Abstract

The entrapment of the ulnar nerve in Guyon’s canal (GC) is a well-known wrist canalicular syndrome which is usually followed by a gradual combination of both sensitive and motor symptomatology. However, GC nerve compression could also cause a pure hand motor dysfunction. This condition, less frequent than the classic Guyon’s syndrome, can be difficult to diagnose. Authors report a case series of eight patients affected by isolated compression of the ulnar nerve motor branch, due to piso-triquetrum or triquetro-hamate joint ganglia. Surgical technique and postoperative outcomes are discussed in this paper. The isolated compression of the ulnar nerve motor branch is a very rare clinical condition which is often linked to several causes. The rarity of the pathology is probably due to lack of knowledge and therefore to the difficulty in formulating a correct diagnosis. Surgical treatment appears to be decisive in most cases, although late diagnosis often leads to incomplete functional recovery.

## Introduction

Entrapment syndromes of the ulnar nerve are quite widespread in the population, second only to carpal tunnel syndrome. The ulnar nerve may be compressed at different points along its course into the forearm and hand. The most frequent site of compression is at the elbow, followed by Guyon’s canal (GC) [1]. The space in which the ulnar nerve runs into the wrist was first described by Felix Guyon in 1861, but it was only in 1908 that a compression syndrome of the ulnar nerve at this level was identified [2, 3]. The etiology of the ulnar nerve compression at the wrist includes soft-tissue tumors, repetitive or acute trauma, the presence of anomalous muscles and fibrous bands, arthritic, synovial, endocrine, and metabolic conditions, iatrogenic injury and soft tissue edema following previous trauma or burns [4]. Depending on whether the compression is at the GC level or further downstream, the symptomatology can be sensitive, functional or a combination of both.

**Fig. 1:**
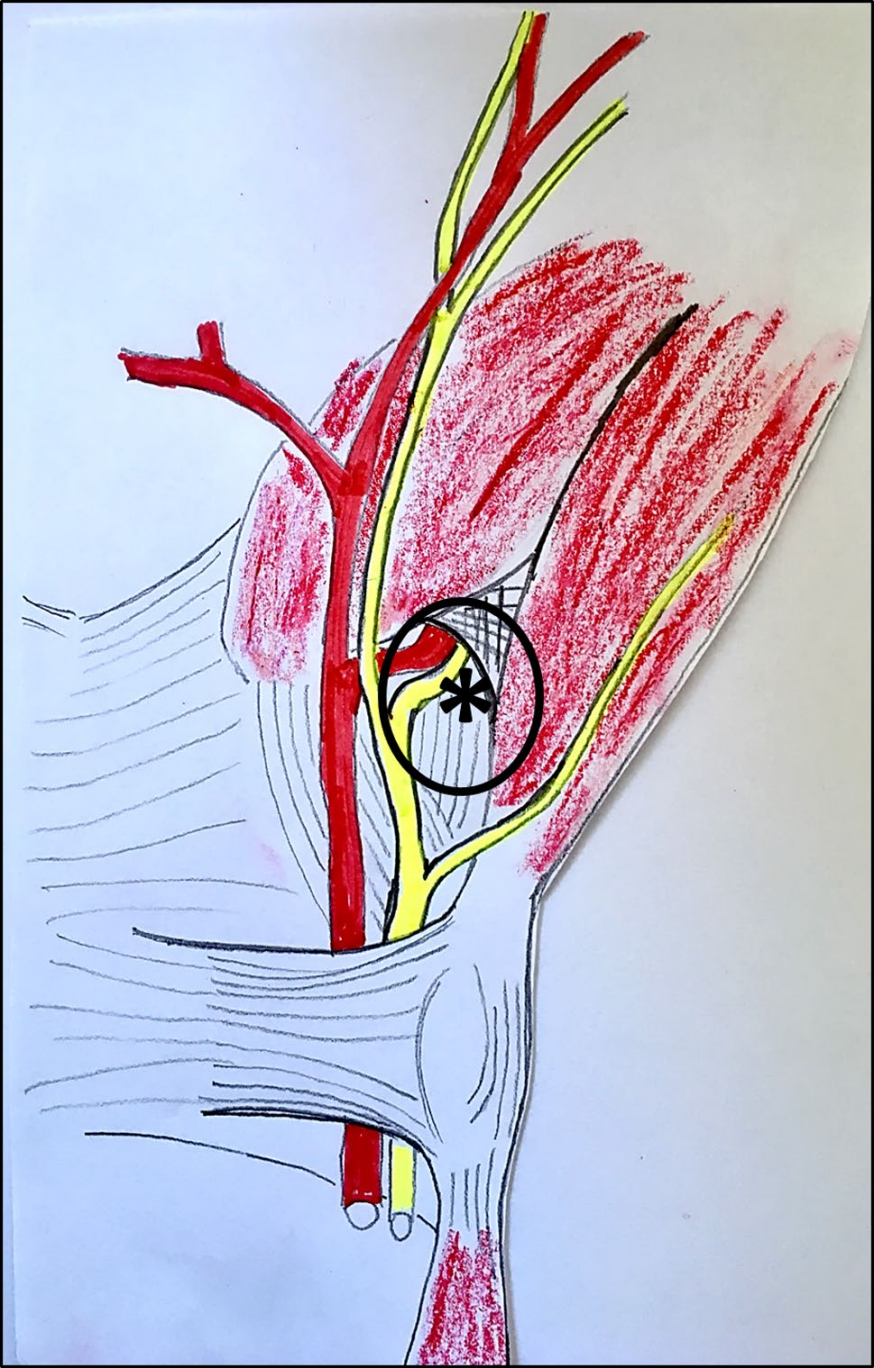
Ulnar nerve crossing the Guyon’s canal as a mixed nerve, then dividing into sensitive and motor branches. The latter runs through the piso-hamate hiatus, site of the ganglion. *critical area

**Fig. 2:**
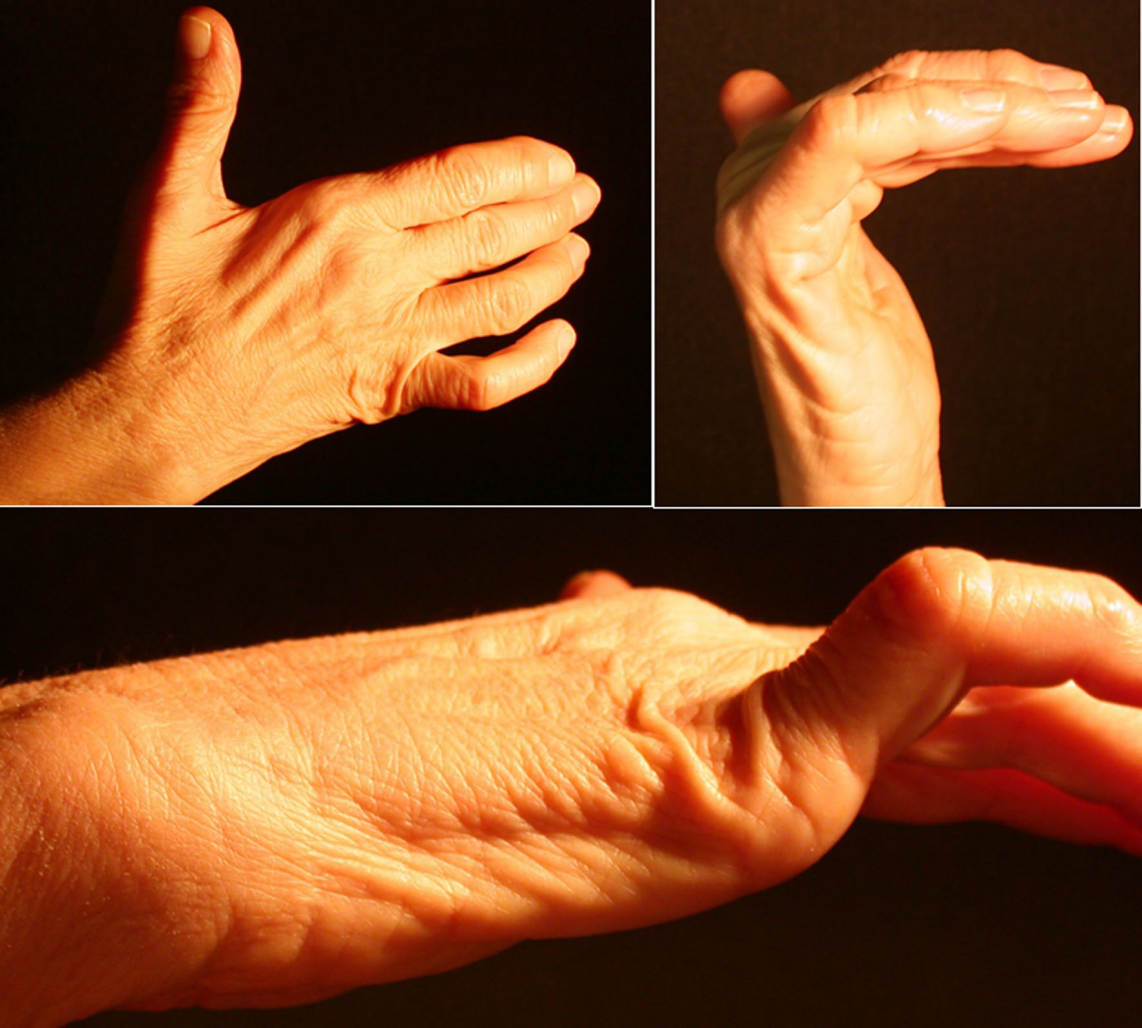
Compression of the motor branch of the ulnar nerve causes weakness and progressive atrophy of the hypothenar muscles, interosseous muscles, third and fourth lumbricals, adductor pollicis, part of the flexor pollicis brevis

The GC is a 4 cm fibrous-bone tunnel. It extends radially-to-ulnary from the transverse carpal ligament, proximally to the pisiform, to the enthesis of the hypothenar muscles at the hook of the hamate. The ulnar nerve passes through the canal and divides into sensory and motor branches. If compression occurs distal to the GC, patients report only a motor dysfunction (Fig. 1). The motor branch then passes through the piso-hamate hiatus and runs transversely as far as the carpometacarpal joints with branches extending into the ulnar interosseous muscles. In addition, at the third metacarpal bone, the motor branch stretches through the adductor pollicis before its terminal branches ramify into the radial interosseous muscles [5]. Should the deep motor branch become affected, only motor symptoms are observed. Typically, the deficit of the muscles innervated by the ulnar nerve determines a claw hand deformity, hypotrophy of the hypothenar eminence and of the first interosseous and of the spaces of the second, third and fourth interossei and adductor pollicis. The isolated compression of the ulnar motor branch enters into differential diagnosis with pathologies of the central nervous system, like multiple sclerosis and amyotrophic lateral sclerosis. Cervical disc herniation, pathologies of the pulmonary apex, as well as systemic pathologies—such as diabetes, amyloidosis, multiple myeloma, alcoholism, etc.—must be excluded. Physical examination is an important step in the diagnosis of this syndrome; however, electrodiagnostic and imaging studies are essential to identify the compressed segments of the nerve. Once the stenosis is confirmed, surgical treatment is crucial. Recovery will depend upon the extent of the compressive damage and the time elapsed between the onset of symptoms and the surgical act; therefore, a delayed diagnosis must be avoided. A pure motor ulnar neuropathy is a very rare condition resulting from the compression of the nerve distal to the GC. This condition was firstly described in 1896 in a cohort of gold polishers [6]. Compression of the motor branch of the ulnar nerve causes weakness and progressive atrophy of the hypothenar muscles, interosseous muscles, third and fourth lumbricals, adductor pollicis, part of the flexor pollicis brevis without sensory dysfunction [7] (Fig. 2). This condition is associated with repetitive traumas or internal sources of compression such as intraneural ganglion, fibrous bands, vessel abnormalities, articular cysts, accessory muscles, fractures of the hook of the hamate bone or hypertrophic scars. Among these, in our clinical experience, articular ganglia are particularly insidious, since their development can either be gradual or sudden, with no previous traumatic events and causing no pain. Furthermore, unlike cysts found in other locations, these ganglia rarely lead to modification of the anatomical profile. In these cases, therefore, the development of motor deficits occurs with no apparent causes, and the diagnosis is often difficult before the onset of motor deficits and muscle denervation, which can be irreversible [8, 9]. We retrospectively report a case series of 8 patients affected by isolated compression of the ulnar nerve motor branch, due to piso-triquetrum or triquetro-hamate joint ganglia. This study has been performed in accordance with the ethical standards and specific national laws have been observed.

## Materials and methods

### Inclusion and exclusion criteria

**Fig. 3:**
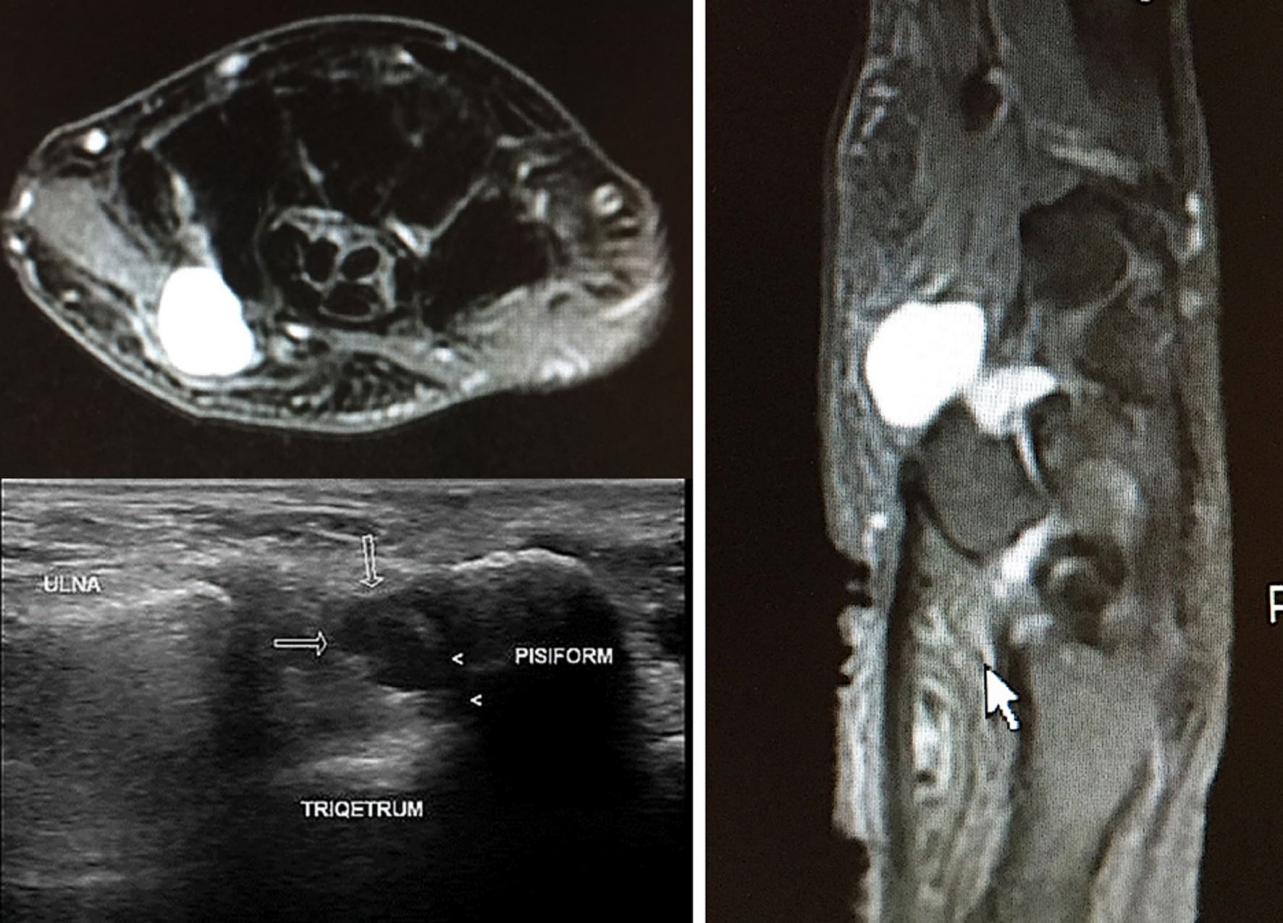
MRI and US of the wrist and hand: huge carpal articular cyst which causes compression of the ulnar motor branch

Starting from a database of 548 patients diagnosed with ulnar nerve compression, surgically treated between 2008 and 2019 in our hospital Hand Surgery Unit, all cases of isolated compression of the ulnar nerve motor branch were identified and collected for our retrospective study. The inclusion criteria were: progressive weakness or paralysis of one or more of the hypothenar muscles and of the adductor pollicis and the flexor pollicis brevis, atrophy of the first interosseus without sensory symptoms, typical electromyographic abnormalities, imaging showing carpal articular ganglion (Fig. 3). Exclusion criteria were: cervical disc herniation, central nervous system diseases, peripheric chronic neuropathies, recent surgery on the wrist and hand. Informed written consent was obtained from all patients included in the study. Clinical examinations, electromyography (EMG)and ultrasound (US) or magnetic resonance imaging (MRI) assessments were performed preoperatively.

### Clinical and imaging assessment

Tests carried out were: Froment test, Wartenberg test, Jeanne test, Masse test and the “Discrimination test of two points” with Dellon Disk, to exclude the presence of sensory deficit [10]. For all the cases, a baseline objective evaluation was recorded using “Patient rated wrist/hand evaluation” (PRWHE) Italian Version [11]. Preoperative and postoperative subjective and objective evaluations were compared. Imaging exams such as US or MRI confirmed the diagnosis in all cases.

### EMG assessment

In addition to routine ulnar nerve motor studies recording the electrical activity from the Abductor Digiti Minimi (ADM) muscle, it is cardinal to study the ulnar motor branch recording the electrical activity from the First Dorsal Interosseous (FDI) muscle and the dorsal ulnar cutaneous sensory nerve 5–8 cm proximal to the wrist. In distal deep palmar motor branch injuries, latency to the FDI may be prolonged with decreased muscle action potential amplitude (CMAP). Also, comparison to the contralateral asymptomatic side is often helpful. When the compression is more proximal, affecting the hypothenar ulnar branches, the distal motor latency to the ADM may also be prolonged. It is mandatory to exclude ulnar neuropathy at the elbow, a lower trunk brachial plexopathy, C8-T1 radiculopathy or motor neuron disease.

Patients were treated surgically by two hand-surgeons and followed-up by two researchers from the same department. Patients underwent the excision of the joint ganglion which caused the compression.

### Surgical Technique

**Fig. 4:**
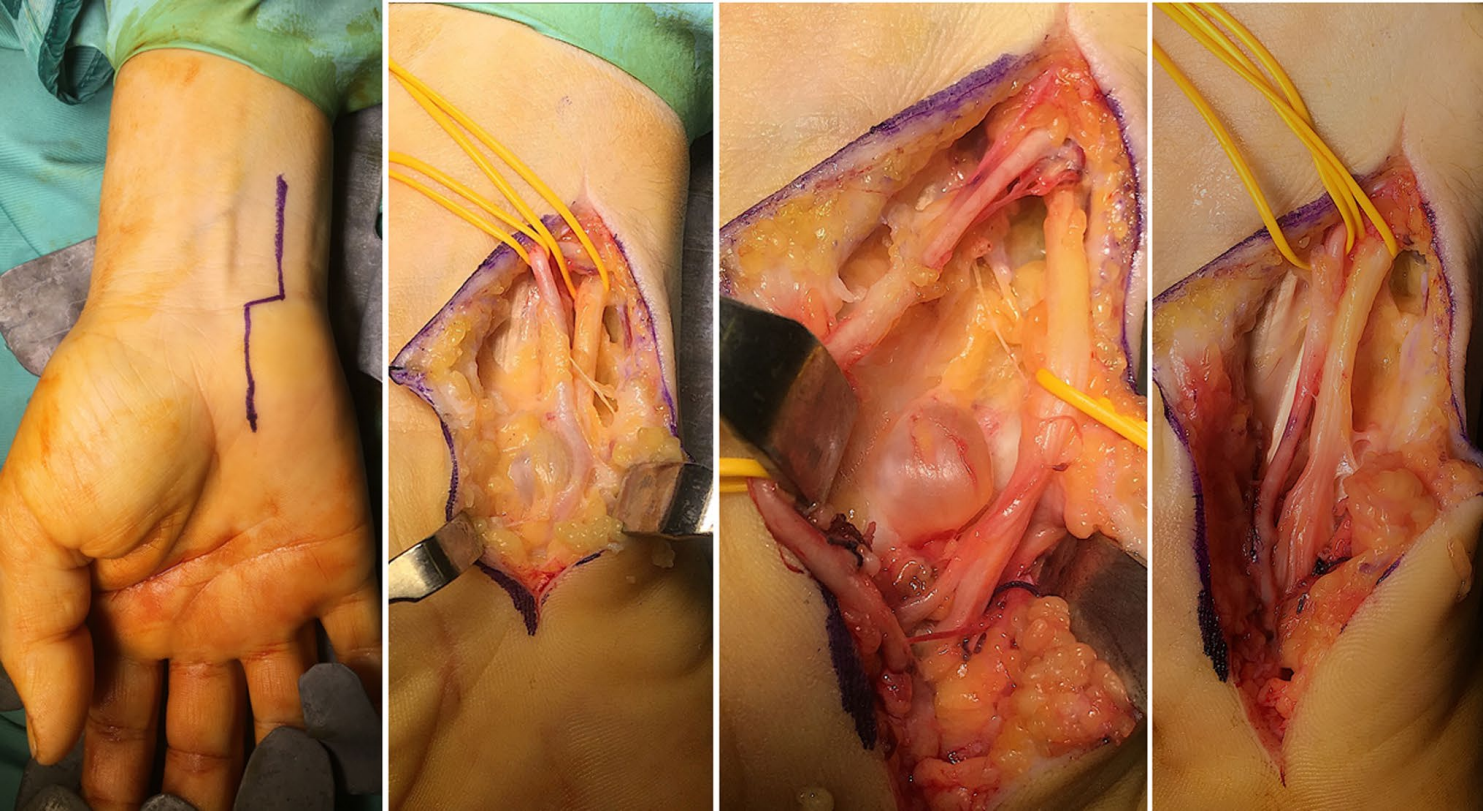
Surgical technique: the incision is longitudinal and radial to the flexor carpi ulnaris tendon, then becomes transversal and again longitudinal over Guyon’s Tunnel. The ulnar nerve and artery are identified, and deeper, the ulnar motor branch. The cause of extrinsic compression is identified and removed

All patients underwent surgery in axillary block anesthesia with a tourniquet to allow good visualization of the surgical field. The incision begins proximal to the wrist, longitudinal and radial to the flexor carpi ulnaris tendon, then the incision becomes transversal at the wrist flexion plica and again longitudinal over the GC, between the pisiform and hamate bones. The fascia is incised radially to the flexor carpi ulnaris tendon, above the ulnar nerve and artery that cross the GC. Two superficial sensitive branches and the deep motor branch of the ulnar nerve are identified. This last insinuates itself vertically under the fibrous arch formed by the abductor of the little finger and the short flexor of the little finger. The ulnar nerve and artery are dissected and protected for the duration of the surgery. The cause of extrinsic compression is identified and removed (Fig. 4). In all cases, an arthrosynovial ganglion caused extrinsic compression on the motor branch of the ulnar from the bottom upwards. The excision was performed taking care to remove the ganglion with its pedicle and its capsule root. After tourniquet release and careful hemostasis, the skin is sutured and a soft dressing is applied for at least 15 days, to allow the patient to move fingers.

### Postoperative care and functional assessment

Postoperatively, patients began a gradual functional recovery, returning to previous activities over 20 days. Clinical outpatient checks were carried out at 15 days, 1 month, 3 months, 6 months and then every 6 months after surgery.

### Statistical analysis

Data were collected in an Excel sheet (Microsoft). A statistical analysis was performed using the SPSS software (IBM Statistics). A comparison of all the variables collected was performed to identify significant differences between the preoperative and the postoperative evaluations. The Student’s *t* test was chosen to study continuous variables. The chi-square test (Fisher exact test) was used to study categorical variables. The confidence interval used was 95% and a value of *p* < 0.05 was considered statistically significant.

## Results

Our retrospective review found 8 patients, 4 males and 4 females. The mean age was 56.5 years (min: 36; max: 78; SD: 12.72) at the time of the surgical treatment. The mean time lapse between the beginning of the symptomatology and surgery was 8.25 months (min: 2; max: 18; SD: 5.49). The mean follow-up was 54 months (min: 12; max: 96; SD: 34.54). All patients showed improved hand function and quality of life as demonstrated by the PRWE score (pre-op: 35.25—min: 12; max: 90; SD: 30.62—post-op: 5.06—min: 0; max: 28.5; SD: 8.43; *p* value < 0.05). Clinical tests were negative in 6 of 8 patients at follow-up. Clinical signs of muscular atrophy (Masse and Wartenberg signs) remained positive in two patients older than 50 years of age, who underwent surgery more than one year after the onset of symptoms, respectively 14 and 18 months (Table 1). Preoperative and postoperative electromyographic evaluations were also conducted. In the preoperatory studies, 3 of 7 patients had prolonged terminal latency to the FDI muscle, all patients had low CMAP of the FDI muscle and normal sensory nerve conduction. Normal needle EMG of the ADM and Abductor Pollicis Brevis  (ABP), increased insertional activity and fibrillation in the FDI were observed in all patients. After surgery, all patients showed a normalization of the distal motor latency values, an increased amplitude of the FDI CMAP and no signs of ongoing denervation (Table 2). One case of painful scar was treated with silicone pads. No iatrogenic injuries or deep infections were observed.

**Table 1:** Demographics and clinical evaluations

Patient	Gender	Age (at the time of surgery)	Elapsed time between diagnosis and surgery	Pre-op physical examination	Post-op physical examination	Pre-op clinical score PRWE	Post-op clinical score PRWE
1	M	56	6 months	4 +	0 +	12	0
2	F	43	2 months	4 +	0 +	15	0
3	M	36	12 months	4 +	0 +	15	0
4	F	59	8 months	4 +	0 +	20	2
5	M	56	18 months	4 +	2 +	30	6
6	M	71	14 months	4 +	2 +	85	28.5
7	F	53	3 months	4 +	0 +	90	0
8	F	78	3 months	4 +	0 +	15	4

The clinical tests used (Froment, Wartenberg, Jeanne, Masse) were positive in all patients before surgery (4 +). Masse and Wartenberg (2 +) signs remained positive in two patients at last follow-up. In these cases, the time elapsed between the onset of symptoms and the surgical procedure was greater than one year. 0 + : all clinical tests were negative at follow-up. All patients showed improved hand function and quality of life as demonstrated by the PRWE score

**Table 2:** Preoperative and postoperative electromyographic evaluations

Patient	Pre-op EMG Abductor Digiti Minimi (ADM)	Post-op EMG Abductor Digiti Minimi (ADM)	Pre-op EMG First Dorsal Interosseous (FDI)	Post-op EMG First Dorsal Interosseous (FDI)
1	2.1 ms 5.8 mV	2.3 ms 6.3 mV	2.5 ms 8.5 mV	2.7 ms 9.2 mV
2	2.5 ms 10.9 mV	2.7 ms 11.8 mV		3.2 ms 13 mV
3	2.3 ms 13 mV	2.6 ms 13.5 mV	2.4 ms 15.6 mV	2.7 ms 16.1 mV
4	1.9 ms 9.1 mV	2.2 ms 9.5 mV	2.4 ms 10.8 mV	2.7 ms 11.1 mV
5	2.8 ms 3.3 mV	2.9 ms 4.5 mV		3.6 ms 6.3 mV
6	2.0 ms 2.7 mV	2.9 ms 4.0 mV	Not evocable	2.8 ms 14.8 mV
7	2.6 ms 6.0 mV	2.9 ms 7.9 mV		3.2 ms 6.6 mV
8	2.1 ms 5.6 mV	2.7 ms 8.1 mV		2.7 ms 10.7 mV

After surgery, all patients showed good distal motor latency values (all values are under 3 ms for the Abductor Digiti Minimi muscle and under 4 ms for the First Dorsal Interosseus muscle) and increased amplitudes for both muscles studied

## Discussion

The purpose of this study is to describe an uncommon ulnar nerve entrapment motor syndrome and the surgical technique to be performed. We wanted to emphasize the need for a timely diagnosis, in order to obtain restoration of motor functions of the intrinsic muscles of the hand. Analysis of the literature has highlighted how few cases describe the sole involvement of the ulnar nerve motor branch. Furthermore, cases of articular ganglion compression are even rarer. This clinical condition may be more frequent than previously imagined. Surgical treatment appears to be decisive in most cases, although late diagnosis often leads to incomplete functional recovery. In fact, the lack of knowledge of this pathology usually leads to hypothesizing pathologies of the central nervous system, delaying treatment for years. In fact, all our patients complained of motor deficits for months and several physicians hypothesized very different etiologies. Causes of compression of the ulnar nerve motor branch can be multiple. Capitani et al. describe a rare case of compression of the motor branch due to repeated microtraumas in a cyclist [12]. In forms related to repeated microtraumas, the work history is of great importance. In fact, manual workers and workers exposed to vibrations are at greater risk of developing this disease. Waugh et al. described motor dysfunction of the ulnar nerve in patients with fractures of carpal bones such as the pisiform, the hamate and the fifth metacarpal base [13]. Even abnormal muscle insertions or fibrous bands can cause compressions as described by Bozkurt et al. [14]. Sometimes, clinical symptoms may be incomplete making the diagnosis even more difficult. This is the case reported by De Maio et al.: a patient presented an isolated paralysis of the adductor muscle of the thumb because of a fibrous band ulnar nerve compression [15]. Diabetes mellitus, alcoholism and renal insufficiency in hemodialytic treatment are related to a higher incidence of ulnar nerve and its terminal motor branch dysfunctions, especially if peri-neural calcium deposits are associated [16]. Jennings et al. describe two other interesting causes of compression of the ulnar nerve motor branch: intraneural ganglion cyst and a constricting leash of vessels [17]. The most frequent cause of ulnar nerve compression, however, is the presence of benign tumors of the soft tissues of the hand such as articular ganglia, giant-cell tumors and lipomas. These can emerge from the ulno-carpal joint, piso-triquetral, triquetro-hamate or other carpal joints [18]. Only a few cases are described in the literature. Gan et al. describe a very peculiar case of a patient with compression caused by both an articular ganglion and lipoma [19]. This underlines the importance of studying every single case in depth before surgical treatment, rather than using just an MRI. In fact, neglecting a cause of compression or not tackling it correctly will significantly compromise an otherwise obtainable result. Wang is the only author to have described a significant number of cases [20]. As in Wang’s nine cases, we also report the compression of the ulnar nerve motor branch alone being caused by an articular ganglion from the piso-triquetrum joint. All the enrolled patients presented a marked improvement in function and strength after surgical decompression. However, Wang's study offers few details of the clinical tests used and their reproducibility. We therefore wanted to objectivize the reported data in the best way possible by using routine and highly reproducible clinical tests and a clinical assessment scale of recognized reliability. Clinical evaluations were repeated by two different hand surgeons experienced in ulnar pathology, in order to optimize the results obtained. This study is unique because, although compression of the motor branch of the ulnar nerve from ganglion is a known etiology, there are not case series in the literature, apart the one by Wang et al. This last, however, is not thoroughly studied. In our experience, systematic clinical and electromyographic assessments made it possible to highlight how an early and accurate surgical treatment allows complete *restitutio ad integrum* of hand function.

The isolated compression of the ulnar nerve motor branch is a low reported clinical condition. The lack of knowledge of this motor neuropathy probably leads to diagnostic difficulties. We believe that a timely clinical and electromyographic evaluation and correct imaging highlighting the cause of compression are crucial in these cases.
